# Numerical Analysis of Cross-Laminated Timber Panels Under Three-Point Bending Using Laminate Theory

**DOI:** 10.3390/ma18225232

**Published:** 2025-11-19

**Authors:** Michal Bošanský, Miroslav Trcala

**Affiliations:** 1 Faculty of Forestry and Wood Technology, Mendel University in Brno, Lesnická 3, 613 00 Brno, Czech Republic; 2 Faculty of Civil Engineering, Brno University of Technology, Veveří 331/95, 602 00 Brno, Czech Republic; miroslavtrcala@seznam.cz

**Keywords:** cross-laminated timber (CLT), bending, laminate theory, finite element method (FEM), three-point bending, structural analysis

## Abstract

Cross-laminated timber (CLT) panels, composed of orthogonally bonded layers, are often used in civil engineering and tall constructions owing to their sustainability, prefabrication advantages and favourable mechanical performance. However, their multilayered, anisotropic and shear-compliant nature presents significant challenges for accurate structural modelling and performance prediction. This study presents an advanced numerical approach to analysing the bending behaviour of CLT panels using the finite element method (FEM) in combination with the classical laminate theory. The proposed plate model was implemented in FlexPDE and validated through a series of three-point bending experiments on three-layer spruce panels. Further verification was conducted using commercial FEM software—Dlubal, incorporating both linear elastic and non-linear damage models, and Abaqus, where a three-dimensional solid model with a cohesive zone formulation captured progressive delamination and local failure in the glued layers. Comparison of the experimental data and numerical simulations revealed strong agreement in load–deflection behaviour, stiffness evolution and damage localisation. The framework we developed accurately reproduces both the global and the local mechanical responses of CLT panels while maintaining computational efficiency. Our results confirm the reliability of laminate theory-based FEM formulations in the design, optimisation and safety assessment of cross-laminated timber structures in building applications.

## 1. Introduction

Engineered cross-laminated timber (CLT) panels have gained increasing attention in multi-storey timber buildings due to their high strength-to-weight ratio, stiffness and sustainable nature. Timber is a renewable material with low embodied energy and carbon sequestration potential, making CLT panels a preferred choice for environmentally responsible construction [1]. Significant advances in CLT technology and construction techniques have been achieved in recent years. However, the lack of comprehensive and up-to-date CLT design standards still poses challenges for the structural design of widely used CLT elements. While methods for analysing light-timber-frame shear walls are included in most building codes and design guidelines, only limited guidance is available on the in-plane stiffness of CLT panels [2]. For example, Eurocodes [3,4] provide only limited information on the design of lateral load-resisting CLT systems in buildings. An engineering-driven approach is needed to develop and refine design methodologies for CLT elements used as primary structural components in tall timber buildings [5].

Recent research has addressed bending, shear and rolling-shear behaviour of CLT panels using various methodologies, including experimental testing, analytical calculations and numerical simulations [6,7,8,9,10,11,12]. Huang et al. (2024) proposed a rolling-shear analysis-based method for determining apparent stiffness and bending capacity of CLT panels under out-of-plane loads [6]. Ply-lam CLT systems, combining timber and plywood layers, have been shown to exhibit modified mechanical behaviour [7], whereas Dobeš et al. (2023) investigated Nordic spruce CLT panels using combined experimental and numerical approaches [8]. Additionally, earlier studies provided fundamental insights into numerical and analytical modelling of timber connections and CLT panel behaviour [10,11], including the influence of in-plane and out-of-plane effects on joint performance [12]. Moreover, alternated cross-laminated timber (aCLT) panels have been developed and mechanically characterised, highlighting innovative laminate configurations and their impact on stiffness and failure [9]. Recent studies also focus on in-plane shear performance of CLT under asymmetric bending tests, providing insight into progressive damage and rolling-shear failure [6].

To allow comparison of different assessment approaches, we modelled a representative bending problem numerically. Our primary model is based on laminate theory, employing two-dimensional shell elements layered together to form a composite CLT panel. A laminate is a composite material made of bonded layers (laminae) and modelled by reference to its homogeneous, linear elastic material properties [10,11,12]. Although CLT is inherently heterogeneous at the microstructural level (timber and adhesive layers), it can be approximated as homogeneous at the macroscopic scale, particularly for linear elastic behaviour in the absence of creep or damage. This assumption relies on the concept of smearing, where adhesive and timber layers are treated as a homogenised continuum.

Timber is modelled as an orthotropic material, with mechanical properties differing in three mutually perpendicular directions. Each unidirectional lamina is orthotropic, and most laminated composites—such as the CLT panels analysed here—fall into this category. The primary material directions are defined relative to the grain orientation of the lamina, which may differ from the principal stress directions as defined in continuum mechanics. CLT panels typically consist of an odd number of laminae arranged symmetrically about the mid-plane, creating a balanced laminate structure [13]. Plies are oriented at 0°, 90°, or other angles depending on panel configuration. The in-plane coordinates of the laminate are denoted as *x* and *y*, with the through-thickness direction as *z*. A plane stress condition is assumed, and the term “plate” is used interchangeably with “composite laminate”, though the laminate may also be regarded as a shell or thin-walled member.

The behaviour of individual plies forms the basis of the laminated plate theory. A key characteristic of anisotropic materials is the presence of coupling effects, which we introduce and apply in the analysis. Subsequently, the relationship between applied loads and resulting deflections is derived. The elastic response of the laminate is described by differential equations and associated constitutive relations. The stacking sequence of plies, a fundamental parameter of laminated composites, is considered, along with derivation of engineering constants—such as stiffness coefficients and compliance matrices—for several laminate configurations. These constants are embedded within the governing equations and are critical for analysing and designing CLT structures under bending and other loading scenarios.

## 2. The Kirchhoff Plate Theory

Normally, stress is defined as the force per unit area applied perpendicular to the cross-sectional surface. The corresponding strain is defined as the elongation per unit length of the material member in the direction of the applied force. The stress–strain relationship under uniaxial loading for isotropic materials, where the material properties are the same in all directions, is governed by a single elastic constant—Young’s modulus.

In contrast, for anisotropic materials such as wood, the elastic behaviour is direction-dependent and must be characterised by multiple elastic constants. In particular, for orthotropic materials (materials with different properties in three mutually perpendicular directions), the stress–strain relationship is described by separate Young’s moduli for the longitudinal and transverse directions within the material plane. In contrast, the stiffness of an orthotropic plate—such as one made from unidirectional-fibre-reinforced wood—must be described using two distinct values: $E_{1}$, the Young’s modulus in the fibre (longitudinal) direction, and $E_{2}$, the modulus transverse to the fibres.

For orthotropic materials, the relationship becomes direction-dependent and can be expressed [13] as follows:

(1) $$\sigma_{1}=E_{1}\cdot \varepsilon_{1},\;\sigma_{2}=E_{2}\cdot \varepsilon_{2}$$

In this formulation, the Kirchhoff plate theory accounts for the direction-specific material behaviour. When applied loads act parallel or perpendicular to the fibres, the plate is considered “specially orthotropic”, meaning its mechanical properties differ in the two in-plane directions but remain constant along each direction.

### 2.1. Assumptions for Orthotropic Materials Based on Hooke’s Law

The main parameter required to solve plane stress problems, linking stress in the longitudinal and transverse directions, is Poisson’s ratio, which is denoted as ν.

Shear forces can also be included via the definition of shear stress and shear strain, which are related through the shear modulus, usually denoted as *G*:

(2) $$\tau_{1,2}=\gamma_{1,2}G_{1,2}$$

Stress as a function of strain is obtained by inverting the compliance matrix:

(3) $$\begin{array}{c} \left[\begin{array}{c} \sigma_{1}\\ \sigma_{2}\\ \tau_{12} \end{array}\right]=\left[\begin{array}{ccc} Q_{11} & Q_{12} & 0\\ Q_{12} & Q_{22} & 0\\ 0 & 0 & Q_{66} \end{array}\right]\left[\begin{array}{c} \varepsilon_{1}\\ \varepsilon_{2}\\ \gamma_{12} \end{array}\right] \end{array}$$

The individual matrix components are as follows:

(4) $$\begin{array}{c} Q_{11}=\frac{E_{1}}{1-\nu_{1,2}\nu_{2,1}},\;Q_{22}=\frac{E_{2}}{1-\nu_{1,2}\nu_{2,1}},\\ Q_{12}=-\frac{\nu_{1,2}E_{2}}{1-\nu_{1,2}\nu_{2,1}}=-\frac{\nu_{2,1}E_{1}}{1-\nu_{1,2}\nu_{2,1}},\;Q_{66}=G_{12} \end{array}$$

The *Q* terms are known as the reduced stiffnesses; the matrix is denoted [Q].

**Figure 1:**
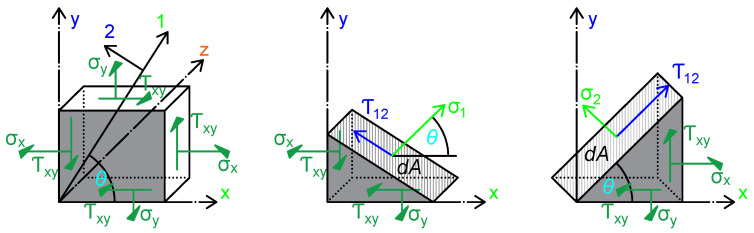
General orthotropic lamina schematic—engineering strain with angle definitions [13].

Stresses and strains in such cases are transformed into components in the principal material directions using the transformation relations illustrated in the domain diagram in Figure 1.

Force balance for shear in the 1–2 plane yields the following:

(5) $$\begin{array}{cc} \sigma_{1} & =\sigma_{x}\cos^{2}\theta +\sigma_{y}\sin^{2}\theta +2\tau_{xy}\sin\theta \cos\theta \\ \sigma_{2} & =\sigma_{x}\sin^{2}\theta +\sigma_{y}\cos^{2}\theta -2\tau_{xy}\sin\theta \cos\theta \\ \tau_{12} & =\left(\sigma_{x}+\sigma_{y}\right)\sin\theta \cos\theta +\tau_{xy}\left(\cos^{2}\theta -\sin^{2}\theta \right) \end{array}$$

This 3×3 transformation matrix, denoted [T], is used in plate theory to transform stress and strain components. Note that the same geometrical considerations apply to the strain transformation using tensorial shear strain. Transformation between the 1,2 and x,y coordinate systems also requires the inverse of [T]:

(6) $$\begin{array}{c} {\left[T\right]}^{-1}=\left[\begin{array}{ccc} \cos^{2}\theta & \sin^{2}\theta & -2\sin\theta \cos\theta \\ \sin^{2}\theta & \cos^{2}\theta & 2\sin\theta \cos\theta \\ \sin\theta \cos\theta & -\sin\theta \cos\theta & \cos^{2}\theta -\sin^{2}\theta \end{array}\right] \end{array}$$

In the next step, the assembly process involves the following:

(7) $$\begin{array}{c} \left[\begin{array}{c} \sigma_{x}\\ \sigma_{y}\\ \tau_{xy} \end{array}\right]={\left[T\right]}^{-1}\left[Q\right]\left[\begin{array}{ccc} 1 & 0 & 0\\ 0 & 1 & 0\\ 0 & 0 & 2 \end{array}\right]\left[T\right]\left[\begin{array}{c} \varepsilon_{x}\\ \varepsilon_{y}\\ \varepsilon_{xy} \end{array}\right] \end{array}$$

The definitions of the new variables *m* and *n* are as follows:

(8) $$\begin{array}{cc} m & =\cos\theta \\ n & =\sin\theta \end{array}$$

The individual components of the lamina stiffness matrix $\bar{Q}$ in terms of *m* and *n* are as follows:

(9) $$\begin{array}{cc} {\bar{Q}}_{11} & =Q_{11}m^{4}+2\left(Q_{12}+2Q_{66}\right)m^{2}n^{2}+Q_{22}n^{4}\\ {\bar{Q}}_{12} & =\left(Q_{11}+Q_{22}-4Q_{66}\right)m^{2}n^{2}+Q_{12}\left(m^{4}+n^{4}\right)\\ {\bar{Q}}_{22} & =Q_{11}n^{4}+2\left(Q_{12}+2Q_{66}\right)m^{2}n^{2}+Q_{22}m^{4}\\ {\bar{Q}}_{16} & =\left(Q_{11}-Q_{12}-2Q_{66}\right)m^{3}n+\left(Q_{12}-Q_{22}+2Q_{66}\right)mn^{3}\\ {\bar{Q}}_{26} & =\left(Q_{11}-Q_{12}-2Q_{66}\right)n^{3}m+\left(Q_{12}-Q_{22}+2Q_{66}\right)nm^{3}\\ {\bar{Q}}_{66} & =\left(Q_{11}+Q_{22}-2Q_{12}-2Q_{66}\right)m^{2}n^{2}+Q_{66}\left(m^{4}+n^{4}\right) \end{array}$$

For angles θ other than zero, the terms ${\bar{Q}}_{16}$ and ${\bar{Q}}_{26}$ become non-zero:

(10) $$\begin{array}{c} \left[\begin{array}{c} \sigma_{x}\\ \sigma_{y}\\ \tau_{xy} \end{array}\right]=\left[\begin{array}{ccc} {\bar{Q}}_{11} & {\bar{Q}}_{12} & 2{\bar{Q}}_{16}\\ {\bar{Q}}_{12} & {\bar{Q}}_{22} & 2{\bar{Q}}_{26}\\ {\bar{Q}}_{16} & {\bar{Q}}_{26} & 2{\bar{Q}}_{66} \end{array}\right]\left[\begin{array}{c} \varepsilon_{x}\\ \varepsilon_{y}\\ \gamma_{xy} \end{array}\right] \end{array}$$

(11) $$\begin{array}{c} \left[\begin{array}{c} \sigma_{x}\\ \sigma_{y}\\ \tau_{xy} \end{array}\right]=\left[\begin{array}{ccc} {\bar{Q}}_{11} & {\bar{Q}}_{12} & {\bar{Q}}_{16}\\ {\bar{Q}}_{12} & {\bar{Q}}_{22} & {\bar{Q}}_{26}\\ {\bar{Q}}_{16} & {\bar{Q}}_{26} & {\bar{Q}}_{66} \end{array}\right]\left[\begin{array}{c} \varepsilon_{x}\\ \varepsilon_{y}\\ \gamma_{xy} \end{array}\right] \end{array}$$

This assembly process shows the coupling between shear strain and normal stresses. Shear strains will produce normal stresses and normal strains will contribute to shear stress. A lamina subjected to a load at an angle other than $0^{\circ }$ or $90^{\circ }$ relative to the fibres exhibits extension–shear coupling. The non-zero terms ${\bar{Q}}_{16}$ and ${\bar{Q}}_{26}$ in the lamina stiffness matrix represent this coupling behaviour.

### 2.2. Mechanical Properties of Laminated Composites

In this section we describe the assumptions we made when analysing the laminated composites. The thickness of the laminate ws assumed to be very small compared to the other dimensions of the plate member.

**Figure 2:**
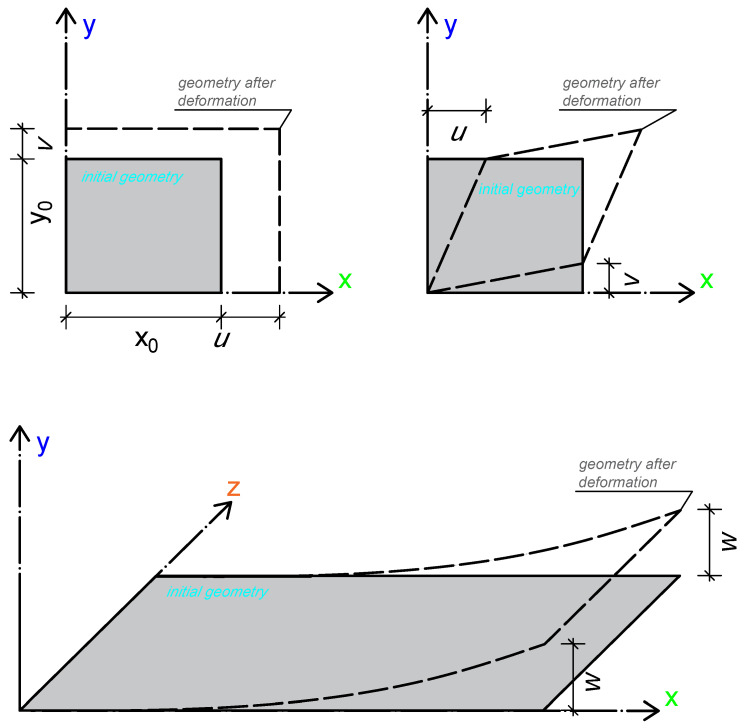
Plate displacement schematics. Normal displacements in a plate are shown in the **upper left** figure, shear displacements in the **upper right** figure and bending displacements in the **bottom** figure [13].

The definitions of displacements in the plate are *u* in the *x* direction, *v* in the *y* direction and *w* in the *z* direction. The relationship between strains and displacements, and the definitions of these displacements, are presented in Figure 2.

**Figure 3:**
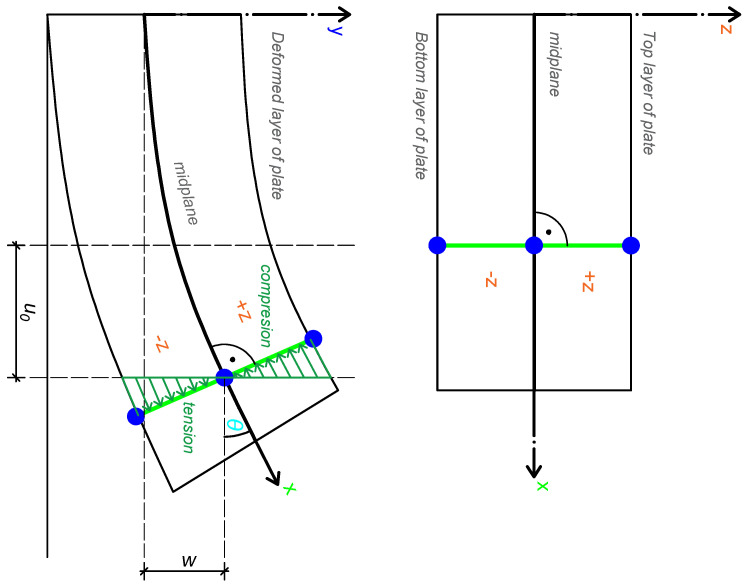
Total plate displacements schematic. The *z*-direction displacement due to bending is sinθ times *z*. Under the condition sinθ≈0, θ is a small angle and the displacement is −z for negative θ (compression) and +z for positive θ (tension) [13].

The sum of the midplane displacements and bending displacements at any reference point represents the total in-plane displacements. The midplane displacements in the plate in the *x* and *y* directions, denoted $u_{0}$ and $v_{0}$, are inputs for determining the total displacements in the plate, as shown in Figure 3. The tangent angle θ, under the small-angle assumption, is defined as $\theta =\frac{\partial w}{\partial x}$.

The total displacements using the midplane displacements in the plate are as follows:

(12) $$\begin{array}{c} u=u_{0}-z\frac{\partial w}{\partial x}\\ v=v_{0}-z\frac{\partial w}{\partial y} \end{array}$$

The classical plate theory neglects strains in the thickness direction and uses only displacements in the thickness direction.

Using equations,

(13) $$\begin{array}{c} \varepsilon_{x}=\frac{\partial u}{\partial x}=\frac{\partial u_{0}}{\partial x}-z\frac{\partial^{2}w}{\partial x^{2}}\\ \varepsilon_{y}=\frac{\partial v}{\partial y}=\frac{\partial v_{0}}{\partial y}-z\frac{\partial^{2}w}{\partial y^{2}}\\ \gamma_{xy}=\frac{\partial u}{\partial y}+\frac{\partial v}{\partial x}=\frac{\partial u_{0}}{\partial y}+\frac{\partial v_{0}}{\partial x}-2z\frac{\partial^{2}w}{\partial x\partial y} \end{array}$$

The initial definitions are as follows:

(14) $$\begin{array}{c} \frac{\partial u_{0}}{\partial x}⟹\varepsilon_{x}^{0}\\ \frac{\partial v_{0}}{\partial y}⟹\varepsilon_{y}^{0}\\ \frac{\partial u_{0}}{\partial y}+\frac{\partial v_{0}}{\partial x}⟹\gamma_{xy}^{0} \end{array}$$

**Figure 4:**
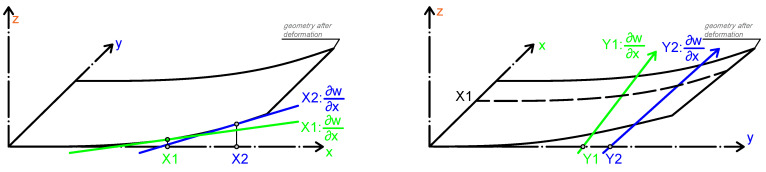
Plate curvature definitions. The rate of change of slope in the *x* direction, $\frac{\partial^{2}w}{\partial x^{2}}=-K_{x}$, is shown in the **left** schematic. The rate of change of slope in the *y* direction, $\frac{\partial^{2}w}{\partial y^{2}}=-K_{y}$, is shown in the **right** schematic [13].

The plate curvature parameters $K_{x}$ and $K_{y}$ describe the change in slope of the bending plate in the *x* and *y* directions, respectively. The parameter $K_{xy}$ describes the change in slope in the *x* direction along the *y* axis. The plate curvature parameters are illustrated in Figure 4.

The stresses in each ply of the laminate can be obtained from Equation (10) using Equation (15):

(15) $$\begin{array}{c} \left[\begin{array}{c} \sigma_{x}\\ \sigma_{y}\\ \tau_{xy} \end{array}\right]=\left[\begin{array}{ccc} {\bar{Q}}_{11} & {\bar{Q}}_{12} & {\bar{Q}}_{16}\\ {\bar{Q}}_{12} & {\bar{Q}}_{22} & {\bar{Q}}_{26}\\ {\bar{Q}}_{16} & {\bar{Q}}_{26} & {\bar{Q}}_{66} \end{array}\right]\left[\begin{array}{c} \varepsilon_{x}^{0}\\ \varepsilon_{y}^{0}\\ \gamma_{xy}^{0} \end{array}\right]+z\left[\begin{array}{ccc} {\bar{Q}}_{11} & {\bar{Q}}_{12} & {\bar{Q}}_{16}\\ {\bar{Q}}_{12} & {\bar{Q}}_{22} & {\bar{Q}}_{26}\\ {\bar{Q}}_{16} & {\bar{Q}}_{26} & {\bar{Q}}_{66} \end{array}\right]\left[\begin{array}{c} K_{x}\\ K_{y}\\ K_{xy} \end{array}\right] \end{array}$$

**Figure 5:**
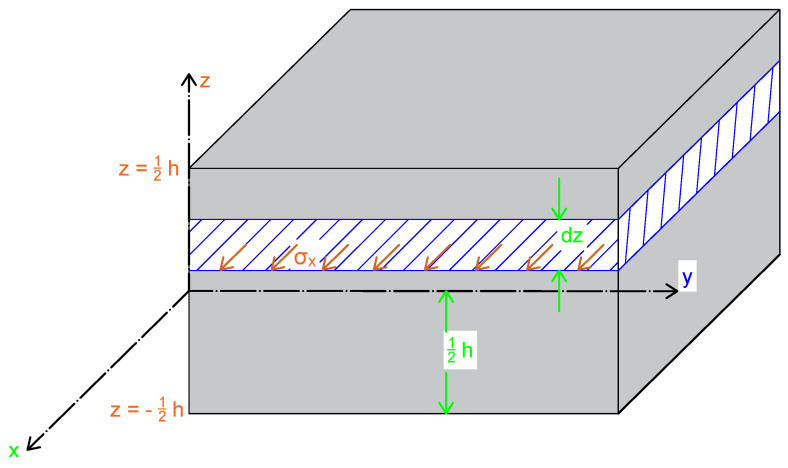
Stress resultant definitions. The total force in the *x* direction is defined as $\sum \sigma_{x}\left(dz\right)$ with dz→0, yielding $\sum \sigma_{x}\left(dz\right)=\int_{-h/2}^{h/2}\sigma_{x}\;dz$. The final definition of the stress resultant as the total force in the *x* direction is $N_{x}\equiv \int_{-h/2}^{h/2}\sigma_{x}\;dz$ [13].

As shown in Figure 5, the total force in the *x*-direction is calculated by integrating the stress $\sigma_{x}$ over the laminate thickness. This definition allows the stress resultant $N_{x}$ to represent the total force per unit width in the *x*-direction.

Although the stress distributions in individual plies vary through the laminate thickness, they can be represented in terms of equivalent forces acting on the mid-surface. The stresses acting on an edge can be divided into increments and integrated to yield the stress resultants, denoted by $N_{i}$, where the subscript *i* indicates the direction. The stress resultant is a force per unit length (line load) acting in the same direction as the stress it represents.

The stresses acting along a line or edge also produce moments relative to the mid-plane of the laminate. The moment resultants depend directly on the distance *z* from the mid-plane.

**Figure 6:**
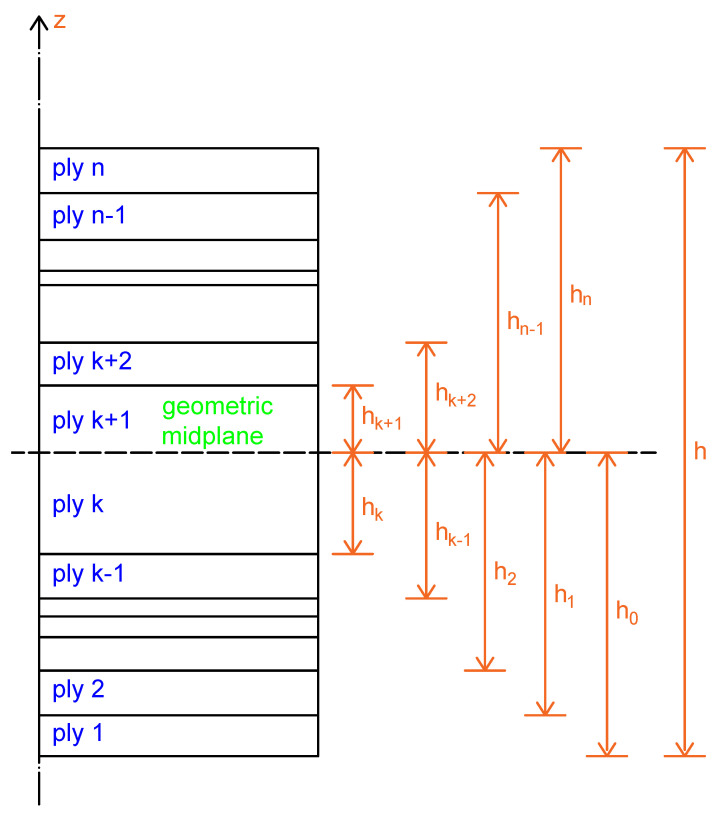
Schematic of individual plies in the cross-section of a laminate. The ply marked *k* and the ply marked k+1 are the same layer, but divided into two plies by the geometric mid-plane of the laminate [13].

The schematic diagram describing the individual plies of a laminate, with the geometric mid-plane indicated, is presented in Figure 6. Discontinuities in stresses may occur at ply interfaces.

Based on the schematic in Figure 6, can be written as follows:

(16) $$\begin{array}{c} \left[\begin{array}{c} N_{x}\\ N_{y}\\ N_{xy} \end{array}\right]=\sum_{k=1}^{n}\int_{h_{k-1}}^{h_{k}}{\left[\begin{array}{c} \sigma_{x}\\ \sigma_{y}\\ \tau_{xy} \end{array}\right]}_{k}dz \end{array}$$

(17) $$\begin{array}{c} \left[\begin{array}{c} M_{x}\\ M_{y}\\ M_{xy} \end{array}\right]=\sum_{k=1}^{n}\int_{h_{k-1}}^{h_{k}}{\left[\begin{array}{c} \sigma_{x}\\ \sigma_{y}\\ \tau_{xy} \end{array}\right]}_{k}z\;dz \end{array}$$

Here, the middle surface strains $\varepsilon^{0}$ and curvatures *K* are independent of *z*. The mid-surface is located at z=0. Because $\varepsilon^{0}$ and *K* are constant through the thickness, they can be taken outside the integrals. The laminate stiffness matrix is constant for a given ply and over its thickness. Moving these strain and curvature vectors outside the integrals and integrating yields the following:

(18) $$\begin{array}{c} \left[\begin{array}{c} N_{x}\\ N_{y}\\ N_{xy} \end{array}\right]=\sum_{k=1}^{n}\left\{{\bar{Q}}_{k}\left[\begin{array}{c} \varepsilon_{x}^{0}\\ \varepsilon_{y}^{0}\\ \gamma_{xy}^{0} \end{array}\right]\left(h_{k}-h_{k-1}\right)+{\bar{Q}}_{k}\left[\begin{array}{c} K_{x}\\ K_{y}\\ K_{xy} \end{array}\right]\left(h_{k}^{2}-h_{k-1}^{2}\right)\right\} \end{array}$$

(19) $$\begin{array}{c} \left[\begin{array}{c} M_{x}\\ M_{y}\\ M_{xy} \end{array}\right]=\sum_{k=1}^{n}\left\{{\bar{Q}}_{k}\left[\begin{array}{c} \varepsilon_{x}^{0}\\ \varepsilon_{y}^{0}\\ \gamma_{xy}^{0} \end{array}\right]\frac{1}{2}\left(h_{k}^{2}-h_{k-1}^{2}\right)+{\bar{Q}}_{k}\left[\begin{array}{c} K_{x}\\ K_{y}\\ K_{xy} \end{array}\right]\frac{1}{3}\left(h_{k}^{3}-h_{k-1}^{3}\right)\right\} \end{array}$$

Because the mid-surface strains and curvatures are not part of the summations, the laminate stiffness matrix and the thickness terms $h_{k}$ can be expressed using new matrices. Equations (18) and (19) define the following:

(20) $$\begin{array}{c} A_{ij}=\sum_{k=1}^{n}{\bar{Q}}_{ij,k}\left(h_{k}-h_{k-1}\right) \end{array}$$

(21) $$\begin{array}{c} B_{ij}=\frac{1}{2}\sum_{k=1}^{n}{\bar{Q}}_{ij,k}\left(h_{k}^{2}-h_{k-1}^{2}\right) \end{array}$$

(22) $$\begin{array}{c} D_{ij}=\frac{1}{3}\sum_{k=1}^{n}{\bar{Q}}_{ij,k}\left(h_{k}^{3}-h_{k-1}^{3}\right) \end{array}$$

The fully partially inverted form of the constitutive equations is given by the following:

(23) $$\left[\begin{array}{c} \varepsilon_{x}^{0}\\ \varepsilon_{y}^{0}\\ \gamma_{xy}^{0}\\ K_{x}\\ K_{y}\\ K_{xy} \end{array}\right]=\left[\begin{array}{cc} A^{\prime } & B^{\prime }\\ B^{\prime } & D^{\prime } \end{array}\right]\left[\begin{array}{c} N_{x}\\ N_{y}\\ N_{xy}\\ M_{x}\\ M_{y}\\ M_{xy} \end{array}\right]$$

with the definition of individual matrix members as follows:

(24) $$\begin{array}{c} \left[\begin{array}{c} A \end{array}\right]=\left[\begin{array}{c} A^{*} \end{array}\right]-\left[\begin{array}{c} B^{*} \end{array}\right]{\left[\begin{array}{c} D^{*} \end{array}\right]}^{-1}\left[\begin{array}{c} C^{*} \end{array}\right] \end{array}$$

(25) $$\begin{array}{c} \left[\begin{array}{c} B \end{array}\right]=\left[\begin{array}{c} B^{*} \end{array}\right]{\left[\begin{array}{c} D^{*} \end{array}\right]}^{-1} \end{array}$$

(26) $$\begin{array}{c} \left[\begin{array}{c} C \end{array}\right]=-{\left[\begin{array}{c} D^{*} \end{array}\right]}^{-1}\left[\begin{array}{c} C^{*} \end{array}\right] \end{array}$$

(27) $$\begin{array}{c} \left[\begin{array}{c} D \end{array}\right]={\left[\begin{array}{c} D^{*} \end{array}\right]}^{-1} \end{array}$$

In the case of symmetric laminates, the configuration about the geometric mid-plane is a mirror image of the ply arrangement above and below the mid-plane. In this case, the geometric mid-plane is also the neutral plane of the plate. For symmetrical laminates, the $\left[\begin{array}{c} B \end{array}\right]$ matrix has all elements equal to zero.

However, for unsymmetrical laminates—where the configuration is not a mirror image above and below the geometric mid-plane—the geometric mid-plane is not the neutral plane of the plate and the $\left[\begin{array}{c} B \end{array}\right]$ matrix will have non-zero elements. To determine the neutral axis of an unsymmetrical laminate, a method based on analysing the stresses within the individual plies is used.

The matrix $\left[\begin{array}{c} A \end{array}\right]$ is called the extensional stiffness matrix. Its elements represent normal stresses and strains (modulus of elasticity). The $A_{12}$ and $A_{16}$ terms couple shear strains to normal stresses and normal strains to shear stresses. For example, when the laminate is subjected to a shear strain, normal stresses will arise, and vice versa. These terms are analogous to the $Q_{12}$ and $Q_{16}$ components introduced earlier in this section.

The matrix $\left[\begin{array}{c} B \end{array}\right]$ in this context is called the coupling stiffness matrix. The constitutive equations show that its elements relate bending strains (plate curvatures) to in-plane normal stresses and vice versa. For instance, the $B_{16}$ and $B_{26}$ terms couple twisting curvatures to normal stresses and shear strains to bending moments.

In symmetrical laminates, the theory assumes that for each ply above the mid-plane, there is a mirrored ply below it (as shown in Figure 6). The $B_{ij}$ elements for plies below the mid-plane (at −z) are equal in magnitude but opposite in sign to those above the mid-plane (at +z). Summing through all plies then yields zero for all $B_{ij}$ terms in a symmetrical laminate.

Finally, matrix $\left[\begin{array}{c} D \end{array}\right]$ is called the bending stiffness matrix and relates the plate curvatures to the bending moments. The $D_{ij}$ terms represent the bending stiffness contributions of the individual plies with respect to the geometric mid-plane of the plate.

### 2.3. Engineering Constants Define the Plane Properties of the Laminate

The in-plane engineering constants of the laminate represent its effective engineering properties. These constants are based on the $\left[\begin{array}{c} A_{ij} \end{array}\right]$ matrix for symmetric laminates, and on the $\left[\begin{array}{c} A_{ij} \end{array}\right]$, $\left[\begin{array}{c} B_{ij} \end{array}\right]$ and $\left[\begin{array}{c} D_{ij} \end{array}\right]$ matrices for unsymmetrical laminates. The orientation of the laminate engineering constants is directly linked to the initial coordinate system introduced.

In the case of a symmetrical laminate, the $\left[\begin{array}{c} B_{ij} \end{array}\right]$ matrix is the zero matrix (i.e., all elements are zero). The determination of the in-plane engineering constants for the laminate then focuses on finding the modulus in the *x* direction.

The simplified constitutive equations for a symmetric laminate, using the fact that the $\left[\begin{array}{c} B_{ij} \end{array}\right]$ matrix is zero, are expressed as follows:

(28) $$\begin{array}{c} \left[\begin{array}{c} N_{x}\\ N_{y}\\ N_{xy} \end{array}\right]=\left[\begin{array}{ccc} A_{11} & A_{12} & A_{16}\\ A_{12} & A_{22} & A_{26}\\ A_{16} & A_{26} & A_{66} \end{array}\right]\left[\begin{array}{c} \varepsilon_{x}^{0}\\ \varepsilon_{y}^{0}\\ \gamma_{xy}^{0} \end{array}\right] \end{array}$$

The Poisson’s ratio of the laminate is as follows:

(29) $$\begin{array}{c} 0=A_{12}\varepsilon_{x}^{0}+A_{22}\varepsilon_{y}^{0}+A_{26}\left(-\frac{A_{16}}{A_{66}}\varepsilon_{x}^{0}-\frac{A_{26}}{A_{66}}\varepsilon_{y}^{0}\right) \end{array}$$

Rearranging (29) gives the following:

(30) $$\begin{array}{c} \nu_{xy}=-\frac{\varepsilon_{y}^{0}}{\varepsilon_{x}^{0}}=\frac{A_{12}-\frac{A_{16}A_{26}}{A_{66}}}{A_{22}-\frac{A_{26}^{2}}{A_{66}}} \end{array}$$

For a symmetrical laminate, this Poisson’s ratio is equivalent to $\nu_{yx}$ due to its orthotropic symmetry.

## 3. Implementation of Plate Theory in Three-Point-Bending

**Figure 7:**
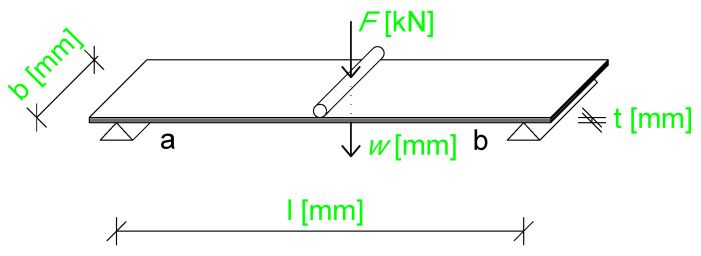
Schematic of the three-point bending test setup. The parameter *l* is the span between the supports, *b* is the width of the plate, *t* is the thickness of the plate represented as the sum of three layers (6/7/6 mm), *F* is the applied force measured by the testing machine at the point of application, and *w* is the deflection measured at the midpoint where the force is applied. In the figure, “a” indicates the location of the support rollers, and “b” denotes the point of force application.

The bending test of the plate sample was performed using a three-point bending setup, as shown in Figure 7.

### 3.1. Application of Kirchhoff Plate Theory Based on Implementing and Defining Differential Relations

Applying the plate theory, we simulated the bending behaviour of a three-layer composite panel using the finite element software FlexPDE v7.20. The specimen consisted of alternating layers of wood and polyurethane adhesive stacked wood–adhesive–wood. The model was built using the classical laminate theory to derive the layer stiffness matrices, which were then assembled into global stiffness terms (A, B, and D matrices).

#### 3.1.1. Geometry and Meshing

The plate dimensions were set to $L_{1}=1.5\;m$ in length and $L_{2}=0.3\;m$ in width. The total thickness was defined as the sum of the thicknesses of the wood and adhesive layers:

$$h=h_{w}+h_{g}=\left(h_{1w}+h_{2w}+h_{3w}\right)+\left(h_{1g}+h_{2g}\right)$$

where typical layer thicknesses were $h_{1w}=6\;\mathrm{mm}$, $h_{2w}=7\;\mathrm{mm}$ and $h_{3w}=6\;\mathrm{mm}$, with optional adhesive layers set to zero in this case.

A structured finite element mesh was generated with a grid density of 10 elements in each direction using cubic basis functions to improve accuracy. An error limit of $10^{-1}$ was used for convergence control.

#### 3.1.2. Material Properties

Orthotropic material properties were assigned to both wood and adhesive layers [14].

Table 1 summarises the linear elastic properties used for the wood and polyurethane adhesive layers. The major and minor Young’s moduli, shear modulus, and Poisson’s ratio for both materials are included. These properties were applied in the finite element model to capture the anisotropic behaviour of the wood and the elastic response of the adhesive layer.

#### 3.1.3. Stiffness Matrices

The reduced stiffness matrices [Q] for each layer were calculated using

$$Q_{11}=\frac{E_{11}}{1-\nu_{12}\nu_{21}},\;Q_{12}=\frac{\nu_{12}E_{22}}{1-\nu_{12}\nu_{21}},$$

$$Q_{22}=\frac{E_{22}}{1-\nu_{12}\nu_{21}},\;Q_{66}=G_{12}.$$

For layers at specific fibre orientations, rotated stiffness terms were calculated using standard transformation equations (Tsai–Pagano formalism), although in this study, we modelled the wood layers with the principal directions aligned with the global axes (0° and 90°).

#### 3.1.4. Global A, B Matrices

The laminate’s extensional (*A*) and coupling (*B*) stiffness matrices were assembled from layerwise contributions, integrated across the thickness:

$$A_{ij}=\sum_{k}Q_{ij}^{\left(k\right)}\left(z_{k}-z_{k-1}\right),$$

$$B_{ij}=\frac{1}{2}\sum_{k}Q_{ij}^{\left(k\right)}\left(z_{k}^{2}-z_{k-1}^{2}\right),$$

where $z_{k}$ denotes the position of the layer interface in the thickness direction. The implementation includes explicit layer-thickness terms and accounts for stacking sequence symmetry.

#### 3.1.5. Loading

Uniform pressure loading was applied, with magnitude

$$p=f+\rho_{w}gh_{w}+\rho_{g}gh_{g}$$

to include the self-weight of the layered construction where *f* denotes the externally applied design pressure load. The model allows for staged loading definitions for non-linear load cases, although linear analysis was performed here.

#### 3.1.6. Solution Strategy

The problem was solved as a static linear bending analysis with in-plane displacements *u* and *v* and out-of-plane deflection *w* as the primary variables. Higher-order derivatives were included to resolve stress and strain gradients. Stability preferences were enabled in FlexPDE, with automatic stage control and error-limiting adaptive refinement.

## 4. Materials and Methods

In this paper, we represented CLT as a solid board composed of three glued layers, a natural material commonly available made from various wood species. The longitudinal outer surfaces consisted of continuous slats glued along the width of the board. The transverse middle layer was made of connected slats. The longitudinal and transverse layers were joined and glued together at a 90-degree angle. The resulting CLT panel has more stability against deflection and twisting than, for example, a solid joint plate. The samples used for the three-point bending experiment were 19 mm thick spruce wood laminates, composed of 6/7/6 mm layers.

Verification of the developed model, based on the classical laminate theory, was carried out using both commercially available software and experimental results. For the software-based verification, we used Dlubal RFEM 6.10 [15], which enables the modelling of layered shell elements to represent cross-laminated timber (CLT) panels. In our case, however, the problem was treated as a two-dimensional task: a vertical cross-section was modelled as a 2D element composed of three layers (three domains corresponding to the layers of the laminated plate). Within Dlubal, we established a case study using a linear orthotropic material model to represent both the timber and the cross-laminated panel. Subsequently, we developed a model incorporating non-linear material behaviour and plasticity. The damage model for the orthotropic timber material was adopted from the literature [16,17,18,19], where concise formulations of the damage model are provided in the context of 2D plane stress conditions.

Table 2 summarises the typical mechanical properties of spruce (C22, 12% moisture content), including linear elastic constants, strength parameters, and non-linear fracture energy properties. The values presented are representative of literature sources [16,20,21,22]. Linear elastic properties, such as Young’s moduli, shear moduli, and Poisson’s ratios, were used to define the orthotropic elastic response of the material. Strength parameters and fracture energies were incorporated to characterise the failure behaviour under different loading conditions.

For the experiments we used a TIRA 2850 S E5 testing machine (TIRA, Schalkau, Germany) with dedicated TIRA VibControl software, available in March 2021. The testing speed was set to 6 ± 0.5 mm/min to ensure that the time to failure did not exceed 90 s, providing a controlled quasi-static loading, in accordance with recommendations from ASTM D198 [23], EN 408:2010 [24] and previous studies on CLT panels [25,26]. The TIRA software allowed precise regulation of the applied force while simultaneously recording the deflection of the specimens throughout the test, enabling a detailed characterisation of their mechanical response. This setup captured not only the initial elastic behaviour but also the progressive onset and evolution of damage leading to ultimate failure. The high resolution and real-time acquisition of both force and displacement data minimised experimental uncertainties and facilitated accurate identification of key mechanical parameters such as stiffness, strength and deformation at failure. The experimental protocol ensured reproducibility across specimens and provided reliable reference data for the validation and calibration of numerical models, allowing for a direct comparison between the observed responses and the predictions of computational simulations.

**Figure 8:**
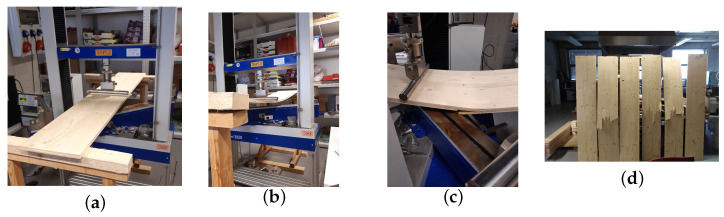
Photographs of the experimental setup and specimens during the three-point bending test: (**a**) view of the testing machine and specimen before loading; (**b**) loaded specimen prior to damage onset; (**c**) loaded specimen with visible damage; (**d**) specimens after the three-point bending test.

We tested nine specimens of three-layer CLT panels under three-point bending (see Figure 8). For each specimen, the load–deflection response was recorded. The numerical simulations were performed using different software tools (FlexPDE, Dlubal, and Abaqus 2021) for the same three-point bending configuration. Due to the limited number of samples, no formal statistical analysis (e.g., ANOVA) was performed. Instead, the mean values and standard deviations of the peak load and mid-span deflection were calculated to evaluate the experimental variability and to compare with numerical predictions. This procedure has been added to the Section 4 for clarification.

## 5. Results and Discussion

**Figure 9:**
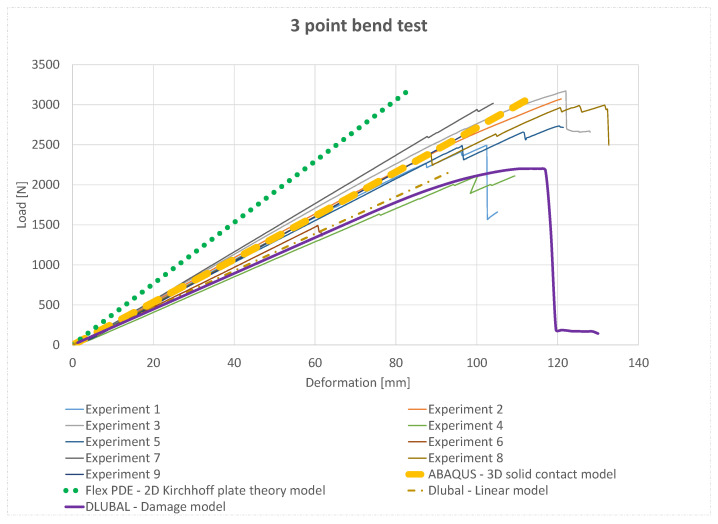
Results of the Kirchhoff plate theory model using FLEX PDE are compared with the experimental samples, and with numerical simulations performed in Dlubal (linear and non-linear material models including damage) and a three-dimensional solid model in Abaqus formulated as a contact problem with defined cohesive properties of the adhesive.

The experimental results, the bending response obtained from the plate model based on the classical laminate theory in FlexPDE, the Dlubal model with linear material properties or incorporating the damage model, and the 3D solid model in Abaqus for three-point bending are presented in Figure 9.

**Figure 10:**
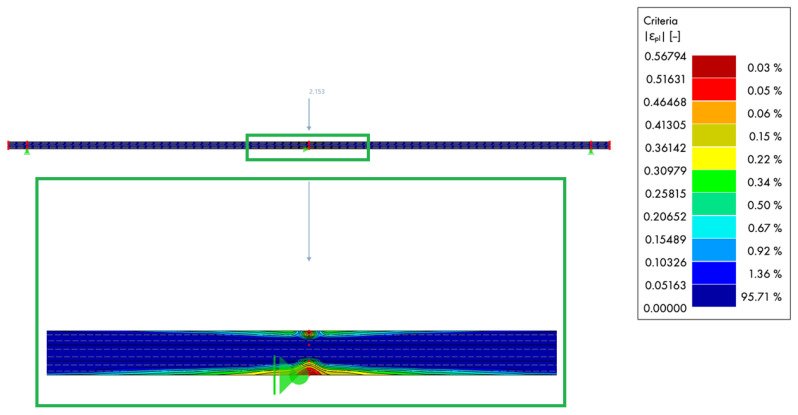
Damage of the beam in the longitudinal x direction during the three-point bending test with a line load of 2153 kN at the beginning of loading.

The damage contour in Figure 10 shows the evolution of the longitudinal (*x* direction) damage variable $D_{x}$ at the onset of loading (*F* = 2153 kN). As expected for a three-point bending configuration, the first activation of $D_{x}$ localises at mid-span on the tensile face, directly beneath the line load where the bending moment is maximal and the fibre-parallel normal stress peaks. Secondary, low-level spots appeared in the shear-dominated regions close to the supports, but remained insignificant in magnitude. The emerging damage bands were aligned with the grain direction, indicating fibre-parallel microcracking, while the compression zone on the top face remained largely elastic at this stage. Overall, the spatial pattern and amplitude of $D_{x}$ were consistent with the predicted stress state and confirm that the model captured the expected sequence of tensile damage initiation before any substantial compressive degradation or shear-induced growth.

The maximum deformation at mid-span provides further insight into the overall model response across different approaches. The plate model with linear material properties predicts deformations closely matching the average behaviour observed in the experimental specimens. The FlexPDE 2D Kirchhoff plate theory model reproduces the general trend of the experiments, although minor deviations occur at higher loads. Incorporating non-linear material behaviour with the damage model in Dlubal resulted in slightly lower mid-span deformations, reflecting stress redistribution and progressive material softening. The Abaqus 3D solid contact model captured both global and local behaviour, including cohesive failure in the glued layers, showing very good agreement with the highest experimental loads. Finally, the linear Dlubal model predicted higher stiffness and load capacity, slightly overestimating them compared to the experimental results. Overall, these comparisons demonstrate that while all numerical approaches capture the general bending behaviour, models incorporating damage or 3D solid representations provide a more realistic prediction of deformation and load-bearing capacity under three-point bending.

## 6. Conclusions

This paper has presented an advanced numerical approach for analysing the bending behaviour of laminated, generally orthotropic, wood-based composite panels subjected to transverse loading. The formulation, grounded in classical laminate theory and implemented within a finite element framework, was validated against experimental three-point bending tests, complementary software simulations and a detailed three-dimensional Abaqus model incorporating a cohesive zone for the glued joints.

The main findings can be summarised as follows:

- The proposed methodology provides accurate predictions of load–deflection behaviour, showing strong agreement with laboratory experiments and 3D Abaqus simulations.
- The modular formulation of stiffness matrices (*A*, *B*, and *D*) enables flexible application to both symmetrical and unsymmetrical laminate configurations while explicitly accounting for coupling effects.
- Comparisons with commercial software, literature-based damage models and cohesive zone simulations confirm the robustness and versatility of the developed framework.
- The approach demonstrates computational efficiency while capturing the essential anisotropic and multilayered nature of laminated boards.

In addition, the spatial distribution of the longitudinal damage variable, captured both numerically and experimentally, confirmed the model’s capability to reproduce the initiation and localisation of tensile failure under bending. The Abaqus 3D solid model further demonstrated the ability to represent local damage in finger-jointed layers and the interaction between timber plasticity and adhesive failure, providing an additional layer of validation for the plate-based approach.

The numerical strategy presented here has the potential to support the design, optimisation and safety assessment of CLT and similar laminated wood-based structures in construction and civil engineering. Future work will focus on extending the framework to non-linear, time-dependent material behaviour, including creep, long-term damage evolution and more complex loading scenarios.

## Figures and Tables

**Table 1:** Orthotropic material properties were assigned to both wood and adhesive layers for wood and polyurethane adhesive layers.

Linear Elastic Properties	Symbol	Value
Wood Young’s modulus	$E_{11}$	10 GPa
Wood Young’s modulus	$E_{22}$	0.33 GPa
Wood Shear modulus	$G_{12}$	0.63 GPa
Wood Poisson’s ratio	$\nu_{12}$	0.35
Polyurethane Young’s modulus	$E_{11}=E_{22}$	22 GPa
Polyurethane Shear modulus	$G_{12}$	0.25 GPa
Polyurethane Poisson’s ratio	$\nu_{12}$	0.44

**Table 2:** Summary of typical mechanical properties of spruce (C22, 12% MC), including linear elastic constants, strength parameters and non-linear fracture energy properties. Values representative of literature sources [16,20,21,22].

Property	Symbol	Value
*Linear elastic properties*		
Longitudinal Young’s modulus	$E_{L}$	9.6 GPa
Radial Young’s modulus	$E_{R}$	1.3 GPa
Shear modulus (L-R)	$G_{LR}$	0.68 GPa
Shear modulus (L-T)	$G_{LT}$	0.60 GPa
Poisson’s ratio (L-R)	$\nu_{LR}$	0.35
Poisson’s ratio (L-T)	$\nu_{LT}$	0.33
*Strengthproperties*		
Tensile strength (parallel to grain)	$f_{t,0}$	33 MPa
Tensile strength (perpendicular to grain)	$f_{t,90}$	3.5 MPa
Compressive strength (parallel to grain)	$f_{c,0}$	37 MPa
Compressive strength (perpendicular to grain)	$f_{c,90}$	4.0 MPa
Shear strength	$f_{v}$	6.9 MPa
*Non-linear(fracture energy) properties*		
Fracture energy (tension, parallel to grain)	$G_{f,0}$	4 N/mm
Fracture energy (tension, perpendicular to grain)	$G_{f,90}$	2 N/mm
Fracture energy (shear)	$G_{f,v}$	1.2 N/mm

## Data Availability

The original contributions presented in this study are included in the article. Further inquiries can be directed to the corresponding author.
